# Safety and Effectiveness of Reduced Dose Versus Standard Dose Enoxaparin Venous Thromboembolism Prophylaxis in Underweight Medically Ill Patients

**DOI:** 10.1177/00185787221123220

**Published:** 2022-09-08

**Authors:** Abigail Nemeth, Manuel Isherwood

**Affiliations:** 1 Penn State Health/Milton S. Hershey Medical Center, Hershey, PA, USA

**Keywords:** anticoagulants, medication safety, adverse drug reactions, cardiovascular

## Abstract

**Background:** Enoxaparin is commonly used for venous thromboembolism (VTE) prophylaxis in hospitalized patients. Published literature exists for dose adjustment in higher body weights and renal dysfunction, but sparse literature on optimal dosing of prophylactic enoxaparin in underweight patients exists. **Objective:** To determine if there is a difference in adverse outcomes or effectiveness if enoxaparin VTE prophylaxis dosing is reduced to 30 mg subcutaneously once daily from standard dosing in underweight medically ill patients. **Methods:** This study was a retrospective chart review of a total of 171 patients, with 190 individual courses of enoxaparin included. Patients were ≥18 years of age, weighed ≤50 kg, and were given at least 2 days of consecutive therapy. Patients were excluded if they were taking anticoagulation upon admission, had a creatinine clearance <30 mL/min, were admitted to the ICU or a trauma or surgical service, or presented with bleeding or thrombosis. The Padua score and a modified score from the IMPROVE trial were used to evaluate baseline thrombotic risk and bleeding risk, respectively. Bleeding events were classified using the Bleeding Academic Research Consortium criteria. **Results:** No difference was seen in baseline risk of bleeding or thrombosis when comparing the reduced and standard dosing groups. No differences were observed with rates of bleeding, thrombotic events, mortality, or 30-day readmission. **Conclusion:** Both reduced and standard dosing strategies appeared effective for VTE prophylaxis, but neither showed superiority in reducing bleeding events. Additional larger studies are needed to evaluate safety and effectiveness of reduced dose of enoxaparin in this patient population.

## Introduction

Enoxaparin is a parenteral anticoagulant that is often used in patients for both prophylaxis for venous thromboembolism (VTE) and therapeutic anticoagulation. Standard dosing of prophylactic enoxaparin for normal weight patients with normal renal function is typically 30 mg subcutaneously every 12 hours or 40 mg subcutaneously every 24 hours.

A variety of studies examining prophylactic enoxaparin dosing have been conducted in the overweight patient population. Freeman et al^1^ conducted a prospective study^1^ that compared anti-Xa levels achieved with three prophylactic enoxaparin dosing strategies in medically ill patients with BMI greater than 40 kg/m^2^. The investigators found that patients in the high dose 0.5 mg/kg subcutaneously daily dosing group achieved target anti-Xa levels more often compared to the 40 mg subcutaneously once daily or the 0.4 mg/kg subcutaneously once daily dosing groups. No increased occurrence of bleeding or thrombosis with the high dose group was observed.

Parikh et al^2^ also examined prophylactic enoxaparin dosing in the overweight population using the 0.5 mg/kg subcutaneously daily or twice daily dosing strategy based on VTE risk. A total of 130 patients were included and 120 patients’ anti-Xa levels were within target range. There was only one bleeding event observed in the study.^2^

Limited literature is available on dosing of prophylactic enoxaparin in the underweight patient population, and current major guidelines^3^ do not comment on the optimal dosing strategy of prophylactic enoxaparin in this population. The aim of this study was to evaluate prophylactic enoxaparin dosing in medically ill patients less than 50 kg by comparing adverse outcomes when dosing is reduced to 30 mg subcutaneously daily from standard dosing of 40 mg subcutaneously daily or 30 mg subcutaneously twice daily.

## Methods

This study was an Institutional Review Board approved retrospective chart review of patients on prophylactic dosing of enoxaparin. Patients who received enoxaparin between March 2015 and December 2015 and between January 2019 and December 2019 were reviewed for inclusion in the study. Inclusion criteria included an age of 18 years or older, a weight of 50 kg or less, and enoxaparin length of therapy of 2 days or more. Patients were excluded if the patient had a bleed or thrombus on presentation for that admission, was on therapeutic or prophylactic doses of enoxaparin prior to or on admission, was on oral anticoagulation or parenteral anticoagulation on admission, was in an intensive care unit, had a creatinine clearance of 30 mL/minute or less, or if the patient was on a surgical or trauma service.

The creatinine clearances used in this study were calculated using the Cockcroft Gault equation. Ideal bodyweight was used if the patient’s actual weight was greater than the ideal bodyweight but not greater than 120%. Actual bodyweight was used if the patient’s actual bodyweight was less than the patient’s ideal bodyweight.

**Figure 1.**
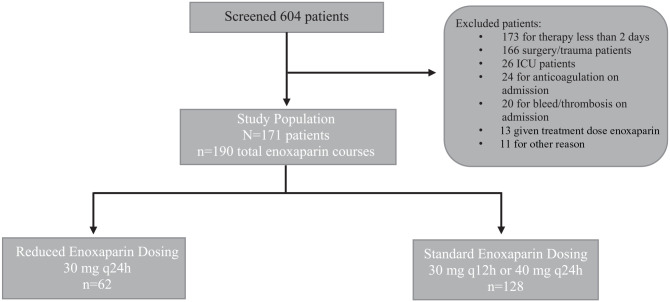
Patient population.

Patient enrollment is described in Figure 1. A total of 171 patients met criteria to be included in the study. Of these patients, 19 patients were on multiple courses of enoxaparin that met inclusion criteria. These patients were eligible to have each separate course counted and assigned to the appropriate treatment group for a total of 190 courses of therapy. The 190 courses of prophylactic enoxaparin therapy were then stratified into 2 groups, a reduced dose group that included patients on enoxaparin 30 mg subcutaneously every 24 hours and a standard dosing group that included patients on 40 mg subcutaneously every 24 hours or 30 mg subcutaneously every 12 hours. The primary outcome of this study was the difference in the number of bleeding events between the 2 groups. Secondary outcomes included differences in rates of thrombotic events, in-hospital mortality, and 30 days re-hospitalization.

Patients included in the study were evaluated for risk of venous thromboembolism (VTE) and bleeding. VTE risk was calculated using the Padua score, with a score of less than 4 points indicating low risk and a score of greater than or equal to 4 points indicating high risk. Bleeding risk was calculated using a modified version of the score used in the IMPROVE trial,^4^ which looked at VTE risk in medically ill patients. The score was modified to exclude the following criteria: hepatic failure, moderate and severe renal failure, ICU patients, and patients who had a central venous catheter. In the IMPROVE trial, a high risk of bleeding was considered a score of greater than or equal to 7 points. With the modifications made, it was estimated an adjusted high risk score was greater than or equal to 4.8 points.

Statistics for this study were conducted using IBM SPSS Statistics Subscription Build 1.0.0.1327. Descriptive statistics were used to analyze the patient demographics. Mann Whitney *U* was used to analyze the Padua score, bleeding risk score, and length of therapy. Fisher’s Exact was used to analyze the remaining outcomes of this study.

## Results

The demographics of the 171 patients included in the study are provided in Table 1. When comparing the reduced dose enoxaparin dosing group to the standard dosing group, the groups were similar in age, weight, gender, and creatinine clearance on admission. Both groups also had a similar length of stay and no difference in length of therapy of enoxaparin (*P* = .471) was observed, indicating patients had similar length of exposure to enoxaparin whether on reduced or standard dosing. Concurrent medications that have been implicated in both increased thrombotic risk (chemotherapy, nicotine in the form of nicotine replacement therapy and smoking, and hormonal therapy) and bleeding risk (antiplatelet agents, non-steroidal anti-inflammatory drugs, steroids) were also collected. The percentage of patients in both groups on concomitant medications with increased risk of thrombosis or increased risk of bleeding were also similar.

**Table 1.** Patient Demographics.

	Reduced dose n = 48	Standard dose n = 123
Age (years), median [IQR]	66 [40,84]	58 [38,75]
Gender-male, n (%)	7 (14.6)	27 (22.0)
Length of stay (days), median [IQR]	5 [3,8]	5 [3,9]
Weight in kg, mean (SD)	43.5 (±5.0)	45.5 (±4.0)
Creatinine clearance, median [IQR]	53.4 [41.3,85.5]	69.4 [51.7,92.1]
Length of therapy (days), median [IQR]	3.3 [1.8,5.4]	3.5 [2.1,6.5]
On medication that increases thrombotic risk, n (%)	10 (20.8)	19 (15.4)
On medication that increases bleeding risk, n (%)	20 (41.7)	68 (55.8)

The Padua score was used to calculate the risk for VTE and the score results can be seen in Table 2. There was no difference seen between the standard dosing group and reduced dosing group in number of patients with high risk of VTE, low risk of VTE, or mean Padua score (*P* = .807). Results of the modified bleeding risk scores can be seen in Table 3. There was no difference in the mean bleeding risk score when the standard dosing and reduced dosing group were compared (*P* = .33).

**Table 2.** Padua Scores.

Patient factor			Score
Active cancer			+3
Previous VTE			+3
Reduced mobility			+3
Thrombophilic condition			+3
Recent trauma/surgery			+2
Age ≥70			+1
Heart and/or respiratory failure			+1
Acute MI/Stroke			+1
Acute Infection and/or rheumatologic disorder			+1
BMI ≥ 30			+1
Ongoing hormonal treatment			+1
	Reduced dose n = 48	Standard dose n = 123	*P* value
High risk	35.4%	37.4%	
Low risk	64.6%	62.6%	
Padua Score, median [IQR]	2 [1,4]	3 [1,4]	*P* = .807

**Table 3.** Bleeding Risk Scores.

Risk factor	Points
Active gastroduodenal ulcer	4.5
Bleeding in 3 months prior to admission	4
Platelets < 50 × 10^9^ cells/L	4
Age ≥85 years	3.5
Hepatic failure (INR > 1.5)	2.5
Severe renal failure GFR < 30 mL/minute	2.5
ICU/CCU	2.5
Central venous catheter	2
Rheumatic disease	2
Current cancer	2
Age 40-84 years	1.5
Male	1
Moderate renal failure GFR 30-59 mL/min	1
Enoxaparin dosing group	Median [IQR]
Reduced dose n = 48	1.5 [1.0,3.75]
Standard dose n = 123	2.5 [1.5,3.5]
	*P* = .33

The primary outcome of bleeding events was classified using the BARC criteria^5^ and the results can be seen in Table 4. There were 14 total bleeding events seen which were broken down into 8 minor bleeds and 6 major bleeds. When comparing the number of minor bleeds ((*P* = .516) and major bleeds (*P* = .362) between groups, there was no difference seen between the 2 dosing groups. There was also no difference in the number of total bleeds between the standard and reduced dosing groups (*P* = .496).

**Table 4.** Bleeding Events.

	Reduced dose n = 62	Standard dose n = 128	*P* value
Minor n (%)	3 (4.8)	5 (3.9)	*P* = .516
Major n (%)	1 (1.6)	5 (3.9)	*P* = .362
Total bleeds n (%)	4 (6.5)	10 (7.8)	*P* = .496

No difference in thrombotic events (*P* = .674), mortality (*P* = .516), and 30 day hospital readmission rates (*P* = .327) was observed when the 2 groups were compared (Table 5). There was one thrombotic event seen during the study time period in a patient in the standard dosing enoxaparin group. The patient was on enoxaparin 40 mg subcutaneously daily and developed lacunar infarcts during the hospitalization. This patient had a Padua score of 6, indicating high risk of developing a VTE. Two patients expired during their hospitalizations, and both were in the standard dose enoxaparin group and on enoxaparin 40 mg subcutaneously daily. A similar percentage of patients also were re-admitted within 30 days of discharge when the 2 dosing groups were compared.

**Table 5.** Mortality and Readmission Rates.

	Reduced dose n (%)	Standard dose n (%)	*P* value
Mortality	0 (0)	2 (1.6)	*P* = .516
Readmission within 30 days	9 (18.8)	18 (14.6)	*P* = .327

## Discussion

At our institution, prophylactic enoxaparin dosing protocols have standard dosage increases at different body mass indexes (BMI) cutoffs for overweight patients with BMIs greater than 35 and 50 kg/m^2^. Our protocol also addresses dose reduction in patients with reduced renal function with a creatinine clearance cutoff of 30 mL/minute, but currently does not include dosing recommendations in underweight patients. This study was conducted to evaluate safety and effectiveness of 2 different dosing strategies, standard and reduced dosing.

To our knowledge, there are limited studies examining dosing of prophylactic enoxaparin in the underweight population. Dybdahl et al conducted a retrospective chart review study that examined enoxaparin VTE prophylaxis dosing in 173 patients weighing less than 45 kg.^6^ The primary outcome was the rate of bleeding events, and the investigators found no difference in major or minor bleeding between patients receiving enoxaparin 30 mg subcutaneously every 24 hours, 30 mg subcutaneously every 12 hours, or 40 mg subcutaneously every 24 hours. They also observed no difference in thrombotic events between the standard and reduced dose groups.^6^ This study did have some limitations which we aimed to address in our study. The authors looked at a very broad patient population and included all levels of care in the hospital as long as a patient had a weight less than 45 kg, was 18 years or older, was not on oral anticoagulation, and had a creatinine clearance of at least 30 mL/minute. The investigators also did not take into account factors that would increase a patient’s chance of developing a thrombosis or bleed such as concomitant medications other than NSAIDs and antiplatelets during admission and if a bleeding or thrombotic event was present on admission. This study also did not evaluate the baseline bleeding risk and thrombosis risk of the enoxaparin dosing groups and if the risk between groups was similar prior to receiving enoxaparin.

Buckheit et al conducted a study examining reduced doses of unfractionated heparin or enoxaparin compared to standard doses for VTE prophylaxis in the inpatient setting.^7^ Their study included 300 patients, of which 131 received enoxaparin at either reduced or standard dose. The primary outcome was the rate of major bleeding. The authors found significantly higher rates of major bleeding in patients who received standard doses of VTE prophylaxis compared to those who received reduced doses. Additional results found no difference in the rates of VTEs or length of hospital stay. While this study improved on prior literature by including IMPROVE scores for enrolled patients to assess baseline risk for VTE, this study did not assess baseline risk for bleeding other than to assess baseline hemoglobin and platelets on admission and the presence of a bleeding disorder or anemia on admission.

Our study found no difference in any of the study outcomes between the standard and reduced dosing groups. This could be due to a combination of multiple factors. It has been postulated that standard dosing of prophylactic enoxaparin may represent an over dosage of enoxaparin in low weight patients as current evidence based on anti-factor-Xa data shows that reduced dosing may better achieve target levels of anticoagulation.^8^ Of the 171 patients enrolled in our study, a greater percentage of patients were receiving standard dosing of enoxaparin VTE prophylaxis. This potentially could have led to an underpowered reduced dosing group. Finally, the possibility exists that in clinical practice, reduced dosing offers no superiority to standard dosing of enoxaparin for clinical outcomes in the underweight population.

Other limitations of our study include the retrospective nature, though we took steps to account for many major confounders which we believed could have impacted the results. Our study relied on data collected by reviewing patient charts, and there could have been inaccuracies or unreported information, particularly regarding minor bleeding events. Finally, we excluded a large number of patients from this study in an effort to remove confounders.

Future research is needed in either the form of larger retrospective studies or meta-analysis of available retrospective data, as well as prospective research.

In conclusion, our study found no difference in safety or effectiveness between reduced dose enoxaparin and standard dose enoxaparin for VTE prophylaxis in the underweight medically ill patient population. Both of these dosing strategies appeared equally effective for preventing VTE.
